# Comparison of methods for deriving phenotypes from incomplete observation data with an application to age at puberty in dairy cattle

**DOI:** 10.1186/s40104-023-00921-5

**Published:** 2023-09-09

**Authors:** Melissa A. Stephen, Chris R. Burke, Jennie E. Pryce, Nicole M. Steele, Peter R. Amer, Susanne Meier, Claire V. C. Phyn, Dorian J. Garrick

**Affiliations:** 1 DairyNZ Ltd, 605 Ruakura Road, Hamilton, 3240 New Zealand; 2 AL Rae Centre for Genetics and Breeding - Massey University, Ruakura, Hamilton, 3214 New Zealand; 3 Agriculture Victoria Research, AgriBio, Centre for AgriBioscience, Bundoora, Victoria 3083 Australia; 4 School of Applied Systems Biology, La Trobe University, Bundoora, Victoria 3083 Australia; 5 AbacusBio, Dunedin, New Zealand

**Keywords:** Cattle, Gibbs sampler, Markov-chain Monte Carlo (MCMC), Puberty

## Abstract

**Background:**

Many phenotypes in animal breeding are derived from incomplete measures, especially if they are challenging or expensive to measure precisely. Examples include time-dependent traits such as reproductive status, or lifespan. Incomplete measures for these traits result in phenotypes that are subject to left-, interval- and right-censoring, where phenotypes are only known to fall below an upper bound, between a lower and upper bound, or above a lower bound respectively. Here we compare three methods for deriving phenotypes from incomplete data using age at first elevation (> 1 ng/mL) in blood plasma progesterone (AGEP4), which generally coincides with onset of puberty, as an example trait.

**Methods:**

We produced AGEP4 phenotypes from three blood samples collected at about 30-day intervals from approximately 5,000 Holstein–Friesian or Holstein–Friesian × Jersey cross-bred dairy heifers managed in 54 seasonal-calving, pasture-based herds in New Zealand. We used these actual data to simulate 7 different visit scenarios, increasing the extent of censoring by disregarding data from one or two of the three visits. Three methods for deriving phenotypes from these data were explored: 1) ordinal categorical variables which were analysed using categorical threshold analysis; 2) continuous variables, with a penalty of 31 d assigned to right-censored phenotypes; and 3) continuous variables, sampled from within a lower and upper bound using a data augmentation approach.

**Results:**

Credibility intervals for heritability estimations overlapped across all methods and visit scenarios, but estimated heritabilities tended to be higher when left censoring was reduced. For sires with at least 5 daughters, the correlations between estimated breeding values (EBVs) from our three-visit scenario and each reduced data scenario varied by method, ranging from 0.65 to 0.95. The estimated breed effects also varied by method, but breed differences were smaller as phenotype censoring increased.

**Conclusion:**

Our results indicate that using some methods, phenotypes derived from one observation per offspring for a time-dependent trait such as AGEP4 may provide comparable sire rankings to three observations per offspring. This has implications for the design of large-scale phenotyping initiatives where animal breeders aim to estimate variance parameters and estimated breeding values (EBVs) for phenotypes that are challenging to measure or prohibitively expensive.

## Background

Time-dependent traits can be logistically challenging and expensive to measure precisely, as animals need to be observed regularly over a long period of time. Some examples include age at puberty (AGEP), mating and calving dates (particularly in the context of beef cattle) and lifespan (which requires culling or mortality dates). In the case of AGEP, indicator traits may be measured repeatedly over a period, with some pre-determined criteria to define an animal as either pubertal or non-pubertal at any given time. Possible measures include behavior monitoring to identify an estrus event, ultrasonography of ovaries to detect the presence or absence of a corpus luteum or testing for elevated blood plasma progesterone (BP4) concentrations that indicate the presence of a functioning corpus luteum [1–3]. Measuring these indicator traits often requires skilled professionals, specialized equipment, facilities, or laboratory resources, as well as a significant commitment from the herd owners who must make their animals available on multiple occasions while somewhat invasive measurements are obtained. These logistical and economic challenges mean that if the AGEP trait is to be measured at sufficient scale for genetic evaluations, it is preferable to establish a phenotype for each animal using as few observations as possible. Therefore, the AGEP trait provides a useful case study of a trait that is rarely measured precisely, and so phenotypes are often subject to censoring.

Past experiments which define the AGEP of individual heifers have measured animals at a range of intervals, including monthly [4], weekly [1] or daily [2, 5]. Researchers should optimize both the length of the observation window and the frequency of measures, according to the cost and effort associated with each additional measure. Optimizing measurement regimes requires assessing the added value of reducing left censoring (animals that are pubertal prior to the start of the observations), interval censoring (animals that become pubertal between two observations), and right censoring (animals that are not pubertal before the end of the observation window).

It is difficult to directly assess the implications of censoring on the genetic analysis of a trait like AGEP, as precise phenotypes are prohibitively difficult to measure at a large-scale. Fortunately, high, positive correlations have been reported between EBVs produced using simulated phenotypes that were either uncensored, or subject to various left-, interval- or right-censoring combinations [6]. Those findings indicate that heritability estimates and EBVs can be robust to phenotype censoring, although this may depend on the methods used to derive AGEP phenotypes from censored observations.

There are several methods that can be used for deriving phenotypes from incomplete observation data which involve converting the incomplete observations into categorical or continuous variables. Researchers often convert censored AGEP observation data into a continuous variable by defining AGEP as the age of the animal when it was first observed to meet the puberty criteria within a set observation period [1, 3, 7]. According to that definition, left and interval censoring are usually ignored, but a penalty approach is often used to handle right censoring. Alternatively, a data augmentation method can be used, where a lower and upper bound are established using incomplete observation data, and an animal’s plausible phenotypes are sampled from a truncated normal distribution using a Gibbs sampling technique [6]. Data augmentation has been used previously to analyze simulated phenotypes subject to right-censoring [8] and left-, interval- and right-censoring [6] with authors reporting only minimal differences between the analysis of censored and uncensored phenotypes. The method used to derive a phenotype from incomplete observations may be important, and a data augmentation approach has been shown to have a slight advantage over other common approaches, particularly for variance parameter estimation [9].

Our primary objective was to investigate the sensitivity of estimated variance parameters and breeding values (EBVs) to varying degrees of observation censoring for a time-dependent trait, using real-life phenotype data. Our second objective was to compare three methods for deriving phenotypes from censored observations. We hypothesized that EBVs and variance parameters would be robust to phenotype censoring, regardless of statistical method. We used AGEP4 as an example trait, but the results of this study may be applicable to many other phenotypes that are derived from incomplete observation data.

## Methods

### Animals

The Ruakura Animal Ethics Committee (Hamilton, New Zealand) approved this study and all manipulations (AE application: 14448). Data were collected from 5,010 dairy heifers, born between July and September 2018 and reared in 54 seasonal calving; pasture-based herds located across three regions (Waikato, Taranaki, Otago) of New Zealand. The average number of heifers from each herd was 88 animals ± 45 (± standard deviation; SD). These 54 herds were selected based on the quality of the existing animal records and predominant breed, with a preference towards herds with mostly Holstein–Friesian animals. The breed proportions for each animal were provided by DairyNZ (Hamilton, New Zealand), and were derived using pedigree records. The resultant study animals were predominantly Holstein–Friesian (i.e., > 90% Holstein–Friesian, *n* = 2,307) or admixed Holstein–Friesian × Jersey crossbred (Holstein–Friesian and Jersey proportions sum to > 90%, but neither Holstein–Friesian or Jersey are > 90% independently, *n* = 2,364), and a small number were predominantly Jersey (> 90% Jersey, *n* = 24). Our analysis also included 50 animals who could not be assigned to the Holstein–Friesian, Jersey or Holstein–Friesian × Jersey breed categories (Other, *n* = 50). Animals with incomplete parentage (*n* = 132), incomplete observations data (*n* = 129), or issues with identification (*n* = 4) were excluded from analysis, leaving 4,745 animals remaining (Table 1). A total of 103 sires were represented by at least 5 daughters.

**Table 1:** Population descriptive statistics of dairy heifers enrolled in age at first blood plasma progesterone elevation phenotype analysis

Breed^a^	No. of animals	No. of herds	Average No. of heifers	No. of sires	No. sires with > 5 daughters
All	4,745	54	88	260	103
HF	2,307	54	43	166	66
J	24	1	24	7	2
XB	2,364	53	45	220	66
OTHER	50	19	3	35	0

^a^Breeds are defined as: > 90% Holstein–Friesian (HF); > 90% Jersey (J); HF + J > 90% but HF < 90% and J < 90% (XB); and HF + J < 90% (OTHER)

### Sampling and measurements

Three sampling visits at approximately 30-day intervals were conducted for each of the 54 herds between May and August 2019. The timing was chosen to meet a target of the animals in each herd being, on average, 327 days old at the second visit, when 45% were predicted to be post-pubertal based up on the stochastic model of Dennis et al. [10]. Accordingly, animals were, on average, 299, 327, and 354 days old (± 14.5) on the first, second and third visits, respectively. At each visit, blood was collected from a coccygeal vessel of animals using blood tubes containing lithium heparin (BD Vacutainers, BD New Zealand, Auckland, New Zealand). Blood samples were immediately placed on ice and were centrifuged (at 4 °C, 1,900 × *g* for 12 min) on the same day as collection. Plasma was separated and stored at −20 °C until BP4 concentration was analysed using a commercial radioimmune assay kit, as previously described [11]. An animal was classified as having elevated BP4 once it had one blood test result indicating a BP4 concentration > 1 ng/mL. This aligns with the criteria previously implemented to characterize onset of puberty in a population of around 500 Holstein–Friesian cows [11].

### Age at first blood plasma progesterone elevation phenotype analyses

We investigated three methods for deriving an AGEP4 phenotype from incomplete observation data. In the analyses presented here, every phenotype was derived from incomplete observations.

Firstly, for the visit category method (CAT), we defined the phenotype for each animal as the consecutive number of the first visit it was observed with BP4 > 1 ng/mL (Table 2). Animals that were observed to have elevated BP4 on the first visit were assigned a score of one (left-censored phenotypes), whereas those first observed with BP4 elevation on the second or third visit were assigned scores of two or three, respectively. Animals with BP4 < 1 ng/mL for the entire trial were assigned a score of four (right-censored phenotypes). We fitted a threshold model to these ordered categorical scores that assumed an underlying normally distributed liability variable, with fixed thresholds that mapped the unobserved liability to the visit score [12]. Sire, rather than animal, was fitted as a random effect, herd was fitted as a fixed effect and breed was fitted as a fixed covariate to estimate variance components. Animal models were not used to analyze phenotypes from this method as convergence failure of the Gibbs sampler is not uncommon for this kind of categorical data [13].

**Table 2:** Example phenotypes (age in day) for animals that have elevated blood plasma progesterone (BP4) or not (Y/N) at each herd visit (one, two or three)

Example animal	A	B	C	D
Actual age at BP4 elevation (AGEP4)	270	380	340	400
Age at visit one (BP4 status visit one)	280 (Y)	350 (N)	300 (N)	330 (N)
Age at visit two (BP4 status visit two)	310 (Y)	380 (Y)	330 (N)	360 (N)
Age on visit three (BP4 status visit three)	340 (Y)	410 (Y)	360 (Y)	390 (N)
CAT	1	2	3	4
AGEVISIT	280	380	360	421
AUG	200–280	350–380	330–360	390–500

A: an animal with elevated blood plasma progesterone at visit one; B: an animal whose BP4 became elevated between visit one and visit two; C: an animal whose BP4 became elevated between visit two and visit three; and D: an animal whose BP4 became elevated after the third visit

CAT: The number of the visit where the animal was first observed with elevated BP4

AGEVISIT: Age (in day) at the visit the animal was first observed with elevated BP4

AUG: Lower and upper bounds of age (in day) when the animal first had elevated BP4

Secondly, we used an age on visit method (AGEVISIT), with the phenotype for each animal defined as its age at the first visit that it was observed with BP4 > 1 ng/mL (Table 2). This phenotype was treated as a continuous variable. Animals with BP4 < 1 ng/mL for the entire trial (right-censored records) were assigned a penalized phenotype of 31 d older than their age on the last visit. A model fitting herd and breed as fixed effects and animal as a random effect was used to analyze this continuous trait [14].

Thirdly, we used a data augmentation method (AUG), whereby the unobserved continuous variable representing actual age at first BP4 elevation was treated as an unknown variable whose value must fall between known upper and lower bounds (Table 2), and plausible values within these bounds were sampled using data augmentation [15]. The upper bound was the age of the animal at the visit it was first observed with BP4 > 1 ng/mL (that is, we knew that they had experienced BP4 elevation on or before this age). The lower bound was the age of the animal at the previous visit (when it had BP4 < 1 ng/mL). For example, the lower and upper bounds of an animal with BP4 > 1 ng/mL on the second visit, would be its age on the first and second visits, respectively. The lower bounds for animals with BP4 > 1 ng/mL on the first visit (left-censored phenotypes) were set to the very young value of 200 days of age, as we would expect all of the animals to be pre-pubertal, with basal BP4 levels at 200-day-old [10]. The upper bounds for animals with BP4 < 1 ng/mL throughout the three visits (right-censored phenotypes) were set to the very old value of 500 d, as we would expect all animals to be post-pubertal, with BP4 elevation by 500-day-old [10]. Plausible AGEP4 phenotypes for each animal were sampled to produce a Markov-chain Monte Carlo (MCMC) posterior distribution for each animal’s AGEP4 based on simultaneous sampling of fixed herd, fixed breed, and random animal effects and variance parameters using single site Gibbs sampling [16].

### Model equation

We fitted a linear model to these data to estimate variance parameters, fixed herd and breed effects, and to obtain EBVs. Matrix representation of the linear mixed model equation is:

1 $${\varvec{y}}={\varvec{X}}{\varvec{b}}+{\varvec{Z}}{\varvec{u}}+{\varvec{e}}$$

where ***y*** is a vector of unobserved liabilities or phenotypes (as defined for each method), ***b*** is a vector of fixed effects, ***u*** is a vector of breeding values (random effects). The vector ***e*** is a vector of residuals corresponding to each of the phenotypes. ***X*** is an incidence matrix relating each phenotype record to relevant fixed effects. All analyses included herd as a fixed effect and proportion Jersey as a fixed covariate. The incidence matrix ***Z*** relates phenotypes to their corresponding EBVs, with a row for each phenotype and a column for each animal represented in ***u***.

### Visit scenarios

**Fig. 1:**
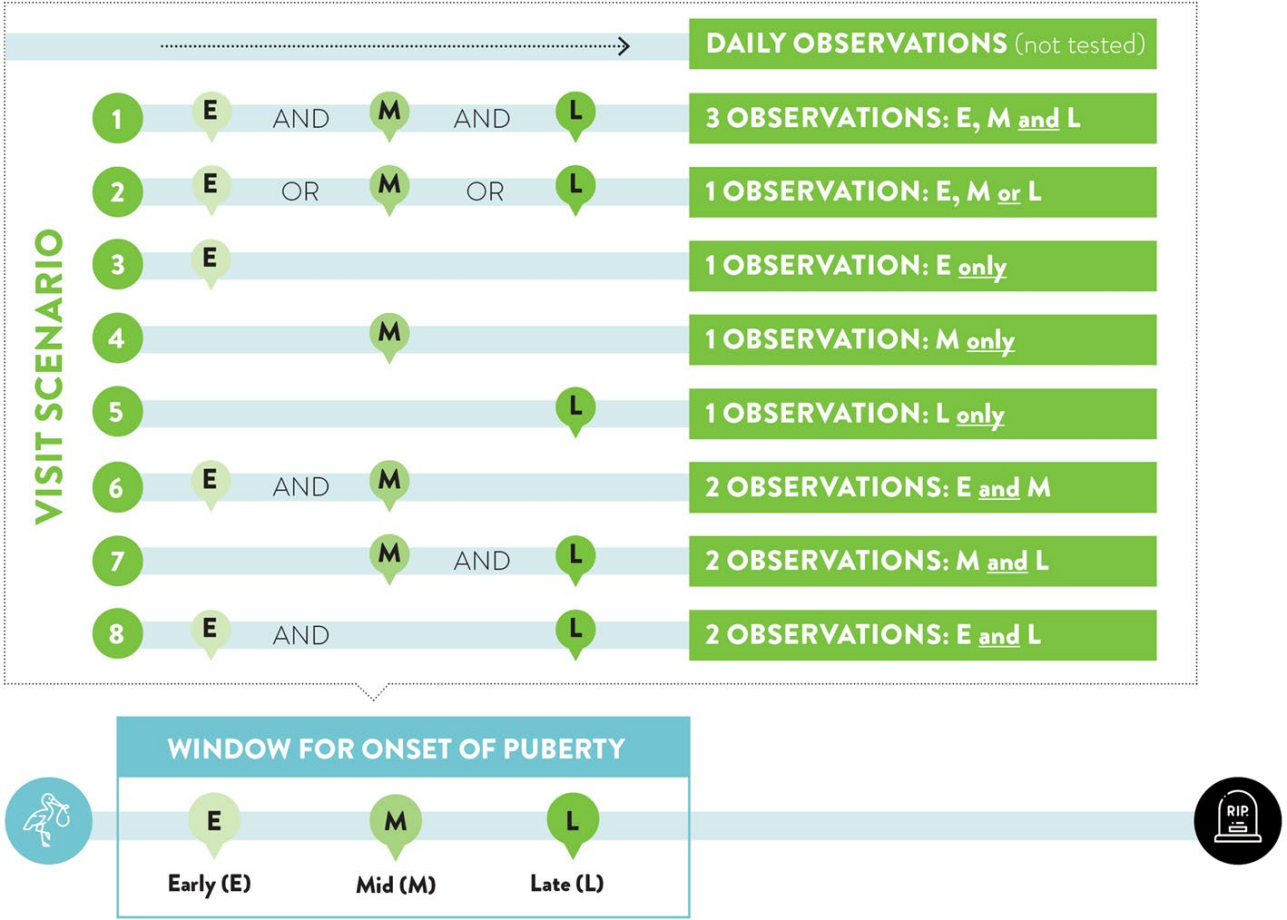
Eight scenarios varying the timing and number of herd visit observations to collect blood progesterone concentrations for measuring age at puberty (AGEP). The first scenario (early, mid, and late; EML) represented the actual experiment comprising of three visit observations. The second scenario (early, mid or late; E/M/L) simulated only one randomly assigned visit observation for each herd. The third, fourth and fifth scenarios simulate that all herds are only visited once E, M or L, respectively. The sixth, seventh and eighth scenarios simulate two observations per herd: early and mid (EM); mid and late (ML); or early and late (EL), respectively

We produced a ‘control’ analysis for each of the three methods tested, where observations from all three visits were used. The results of these control analyses were then compared (within method) with results of seven alternate test visit scenarios. Test visit scenarios varied in timing or frequency of the observations that were retained. Each test scenario had a proportion of data selectively excluded to alter left, right, or interval censoring. In total, we defined 8 scenarios (Fig. 1). The first scenario (early, mid, and late; EML) represented the actual experiment comprising of three visit observations. The second scenario (early, mid or late; E/M/L) simulated only one randomly assigned visit observation for each herd. The third, fourth and fifth scenarios simulated that all herds were only visited once and that visit is either E, M or L, respectively. In these visit scenarios (2 to 5) all phenotypes were subject to either left or right censoring, and the ratio was varied depending to which visit was included. The sixth, seventh and eighth scenarios simulated two observations per herd: early and mid (EM); mid and late (ML); or early and late (EL), respectively. In these visit scenarios (6 to 8) all phenotypes were subject to either left-, interval- or right-censoring. The ratio of phenotypes that were subject to left-, interval- or right-censoring varied depending on which visits were included.

### Software and solver

We used command line bash scripts to pre-process observation data and produce files containing the phenotypes for each method and visit scenario combination. We performed the genetic analysis and post-processing using the JWAS package [17] implemented in Julia [18]. A MCMC technique was applied using a single site Gibbs sampler to obtain samples from the posterior distributions for fixed and random effects, variance parameters, and in the case of the AUG method, plausible phenotypes within the known lower and upper bounds for each animal. The MCMC comprised 100,000 samples of every unknown, with the first 50,000 samples disregarded as a burn-in. The Julia packages CSV, StatsPlots, DataFrames were used to post-process the results. We assessed MCMC convergence by grouping post burn-in samples consecutively in lots of 10,000 (group one = sample 50,000 to 60,000, group two = sample 60,000 to 70,000 etc.) and comparing the mean and distribution of sample groups. The models were considered to have converged when the 95% credibility intervals consistently overlapped across groups.

We produced 95% credibility intervals that were thresholds for the 2.5% (lower bound) and 97.5% (upper bound) percentile of all values samples within the MCMC. That is, 95% of the plausible values fell within the credibility intervals presented.

### Criteria for comparison

We used the stability of estimated variance parameters, breeding values (EBVs) and fixed effect solutions across varying degrees of phenotype censoring to assess the sensitivity of these parameters to increased phenotype censoring. That is, if a given parameter was impervious to increased phenotype censoring, we would expect no change in estimated variance parameters, breeding values (EBVs) and fixed effect solutions as phenotype censoring was increased. To assess stability of estimated variance parameters we compared the posterior mean and 90CRI of heritability estimates. Similarly, we also compared the posterior mean and 90CRI of fixed breed covariate solutions. To assess the stability of EBVs and fixed herd effect solutions we calculated the Pearson correlation coefficient between EBVs produced using our control scenario (early, mid and late visits included) and the EBVs produced using each test scenario. A higher correlation demonstrated greater stability.

## Results

### EBV correlations between visit scenarios

Correlations between sire EBVs (sires with > 5 daughters, *n* = 103) from the control scenario (EML) and scenarios E/M/L, E, M, L, EM, ML, and EL were positive and mostly high (CAT* r* = 0.67 to 0.93, AGEVISIT *r* = 0.65 to 0.95, AUG *r* = 0.75 to 0.95) (Table 3). Correlations decreased as phenotype censoring increased. The sire EBVs produced using the E/M/L scenario (which had just one randomly timed visit per herd) exhibited the lowest correlation with sire EBVs produced using the control scenario.

**Table 3:** Correlations between estimated breeding values (EBVs) from scenario one relative to those from scenarios two to eight for the three different methods used to derive age at first blood plasma progesterone elevation (AGEP4) phenotypes

Scenario		Correlations between EBVs relative to scenario one (EML)		
		CAT	AGEVISIT	AUG
1	Early, mid and late visit (EML)	1.00	1.00	1.00
2	Early, mid or late visit (E/M/L)	0.67	0.65	0.75
3	Early visit only (E)	0.83	0.69	0.87
4	Mid visit only (M)	0.76	0.74	0.83
5	Late visit only (L)	0.74	0.75	0.83
6	Early and mid visit (EM)	0.94	0.95	0.95
7	Mid and late visit (ML)	0.90	0.95	0.94
8	Early and late visit (EL)	0.93	0.94	0.95

CAT: The number of the visit where the animal was first observed to have elevated BP4; AGEVISIT: Age in day at the visit the animal was first observed to have elevated BP4; and AUG: The continuous variable AGEP4 sampled from between the known lower and upper bounds. Correlations include within breed EBVs for sires that had > 5 daughters with an AGEP4 phenotype (*n* = 103). Scenarios are described in Fig. 1, and analysis methods are described in Table 2

Among the methods, the AUG method generally had the highest correlations between the control scenario and the other scenarios. For scenarios E, M and L when there was a single visit to each herd, correlations ranged from *r* = 0.74 to 0.83 for the CAT method, *r* = 0.69 to 0.75 for the AGEVISIT method, and *r* = 0.83 to 0.87 for the AUG method. In contrast, for scenarios EM, ML and EL, when there were two visits to each herd, there were little differences across methods between the correlations with the control EML scenario (*r* = 0.90 to 0.95).

### Breed effect

We estimated the breed effect for each analysis (Table 4) as the difference in AGEP4 between Jersey and Holstein–Friesian animals. For the CAT and AUG methods, the breed difference was consistently negative (CAT on the one to four categorical scale: −1.67 to −0.84, AUG: −56 to −25 d); whereas for the AGEVISIT method, the credibility interval for breed difference spanned zero for the four scenarios based upon one herd visit (AGEVISIT: −29 to 1 d). For all methods, the size of the breed difference tended to decrease as phenotype censoring increased.

**Table 4:** Breed (Jersey relative to Holstein–Friesian) effect solutions and 95% credibility intervals from scenarios one to eight, for the three different methods used to analyze age at first blood plasma progesterone elevation (AGEP4) phenotypes

Scenario		Breed (Jersey) effect solution		
		CAT	AGEVISIT	AUG
1	Early, mid and late visit (EML)	−1.62 (−1.26,−1.99)	−29 (−15,−43)	−56 (−34,−79)
2	Early, mid or late visit (E/M/L)	−1.31 (−0.87,−1.75)	0 (7,−7)	−40 (−18,−62)
3	Early visit only (E)	−1.58 (−1.09,−2.06)	−2 (5,−10)	−47 (−24,−72)
4	Mid visit only (M)	−1.47 (−1.01,−1.92)	−4 (3,−11)	−45 (−23,−67)
5	Late visit only (L)	−0.84 (−0.44,−1.26)	1 (8,−7)	−25 (−5,−44)
6	Early and mid visit (EM)	−1.67 (−1.26,−2.06)	−17 (−7,−27)	−54 (−34,−74)
7	Mid and late visit (ML)	−1.46 (−1.07,−1.85)	−16 (−6,−26)	−45 (−26,−64)
8	Early and late visit (EL)	−1.45 (−1.07,−1.83)	−24 (−11,−37)	−54 (−28,−78)

CAT: the number of the visit where the animal was first observed to have elevated BP4; AGEVISIT: age in day at the visit the animal was first observed to have elevated BP4; and AUG: the continuous variable AGEP4 sampled from between the known lower and upper bounds. Scenarios are described in Fig. 1, and analysis methods are described in Table 2

### Herd effects

We also estimated herd effects for each analysis (Table 5) based upon the expected phenotype of an average merit animal in that herd, where other fixed effects (in this case, proportion Jersey) were zero. Herd effect (*n* = 54) correlations between the control EML scenario and visit scenarios (E/M/L, E, M, L, EM, ML and EL) were positive and generally high across methods (CAT *r* = 0.45 to 0.99, AGEVISIT *r* = 0.15 to 0.98, AUG *r* = 0.87 to 0.98). For all three methods, herd effect correlations decreased as phenotype censoring increased. The correlations of herd effect solutions between the control scenario and scenarios E, M and L, when only one visit was included per herd, were highest for the AUG method (CAT *r* = 0.45 to 0.93, AGEVISIT *r* = 0.15 to 0.80, AUG *r* = 0.87 to 0.95). The correlations of herd effect solutions between the control scenario and scenario E/M/L (which had just one randomly timed visit per herd) were lowest for the AGEVISIT method (*r* = 0.15), and highest for the AUG method (*r* = 0.87). For scenarios EM, ML and EL, when two visits were included, the correlations of herd effect solutions from the control scenario were generally very high (CAT *r* = 0.51 to 0.99, AGEVISIT *r* = 0.96 to 0.98, AUG *r* = 0.97 to 0.99).

**Table 5:** Correlations between herd (*n* = 54) effect solutions from scenario one relative to those from scenarios two to eight for the three different methods for analysing age at first blood plasma progesterone elevation (AGEP4) phenotypes

Scenario		Herd effect correlations relative to scenario one (EML)		
		CAT	AGEVISIT	AUG
1	Early, mid and late visit (EML)	1.00	1.00	1.00
2	Early, mid or late visit (E/M/L)	0.47	0.15	0.87
3	Early visit only (E)	0.45	0.76	0.87
4	Mid visit only (M)	0.53	0.80	0.95
5	Late visit only (L)	0.93	0.76	0.93
6	Early and mid visit (EM)	0.51	0.97	0.98
7	Mid and late visit (ML)	0.97	0.96	0.97
8	Early and late visit (EL)	0.99	0.98	0.99

CAT: the number of the visit where the animal was first observed to have elevated BP4; AGEVISIT: age in day at the visit the animal was first observed to have elevated BP4; and AUG: the continuous variable AGEP4 sampled from between the known lower and upper bounds. Scenarios are described in Fig. 1, and analysis methods are described in Table 2

### Heritability

The credibility intervals for heritabilities overlapped by scenario (rows) and methods (columns), as presented in Table 6. Nevertheless, the CAT method tended to produce the highest heritabilities across all scenarios, whereas the AGEVISIT method produced the lowest heritabilities, with intermediate heritabilities resulting from the AUG method. Furthermore, heritabilities tended to be higher when scenarios included the ‘early’ observation (i.e., EML, E, EM, or EL scenarios).

**Table 6:** Heritabilities and 95% credibility intervals from scenarios one to eight for the three different methods used to analyze age at first blood plasma progesterone elevation (AGEP4) phenotypes

Scenario		Heritability		
		CAT	AGEVISIT	AUG
1	Early, mid and late visit (EML)	0.39 (0.24,0.58)	0.23 (0.16,0.32)	0.32 (0.21,0.46)
2	Early, mid or late visit (E/M/L)	0.23 (0.13,0.38)	0.19 (0.13,0.28)	0.21 (0.11,0.35)
3	Early visit only (E)	0.36 (0.20,0.58)	0.29 (0.20,0.40)	0.31 (0.14,0.54)
4	Mid visit only (M)	0.23 (0.12,0.39)	0.19 (0.13,0.27)	0.19 (0.10,0.33)
5	Late visit only (L)	0.20 (0.10,0.35)	0.16 (0.10,0.22)	0.14 (0.05,0.26)
6	Early and mid visit (EM)	0.40 (0.24,0.62)	0.22 (0.15,0.30)	0.30 (0.19,0.45)
7	Mid and late visit (ML)	0.26 (0.15,0.42)	0.17 (0.11,0.23)	0.23 (0.11,0.36)
8	Early and late visit (EL)	0.33 (0.19,0.50)	0.19 (0.12,0.26)	0.28 (0.17,0.41)

CAT: the number of the visit where the animal was first observed to have elevated BP4; AGEVISIT: age in day at the visit the animal was first observed to have elevated BP4; and AUG: the continuous variable AGEP4 sampled from between the known lower and upper bounds. Scenarios are described in Fig. 1, and analysis methods are described in Table 2

## Discussion

### Sensitivity of sire EBV rankings to increased censoring depended on analysis method

In this study, we quantified the extent of sire re-ranking based on their AGEP4 EBVs across scenarios with different phenotype censoring using a Pearson correlation coefficient (Table 3). We determined that the sensitivity of sire re-ranking to increased censoring varied across the three analysis methods investigated. When there was large re-ranking of sires between scenarios, it follows that sire selection and thus genetic progress [19] will depend on the timing and frequency of observations. We determined that of the three analysis methods explored, the AUG method provided the most robust EBVs across the eight scenarios tested.

Sire EBV rankings were robust (*r*  ≥ 0.90) for all methods between our control scenario (EML) and scenarios with two observations included (i.e., EM, ML, and EL). These high correlations indicate that sire selections would be similar if offspring had two or three observations, and, in general, those two observations can be any combination of the early, mid-point or late herd visits. Our results indicate that for the purpose of determining sire rankings for genetic selection, there may be limited marginal gain in a third observation per animal. This finding could be useful for large-scale phenotype collection for routine genetic evaluations or for future trial design, as AGEP and other time-dependent binary traits can be logistically and economically difficult to measure.

The marginal gain of a second visit appears to be higher than that of a third visit, as greater re-ranking was apparent across all methods when scenarios included only one visit (i.e., E/M/L, E, M, or L). However, the extent of EBV re-ranking depended on the analysis method; EBVs produced by the AUG method were the most robust to this degree of phenotype censoring based upon a single observation. Furthermore, the timing of the single visit affected sire re-ranking. For the CAT and AUG methods, scenario E based on a single ‘early’ visit per herd resulted in the least re-ranking relative to our control scenario. In general, the AGEVISIT method resulted in the most re-ranking when only a single visit was included, but the timing of a single visit did not appear to be as important. These results suggest that EBVs calculated using the AUG method are robust using a single observation per animal, potentially reducing the data measurement effort required and improving the scalability of phenotype collection. For example, a single observation per animal could enable a larger number of animals to be measured, which would improve the accuracy of sire EBVs, increasing the number of selection candidates, and thus the selection intensity for the trait.

Furthermore, the E/M/L scenario represents a likely practical measurement regime should a phenotype like AGEP4 be measured at scale. Seasonal calving dates tend to be aligned within regions of New Zealand and other countries that have pasture-based dairy systems, which means that the average ages of birth year groups are similar across herds. Hence, it may be infeasible to collect heifer BP4 status across large numbers of herds at a specific average age. Instead, it would be more reasonable to recommend that animals are measured on a single visit within a defined age window. We were not able to fully test this scenario using our current data, as our visits were scheduled to occur at only three average herd ages (297, 327 and 357 d). However, if this phenotype was measured at scale, we would likely obtain BP4 status for a sire’s offspring across a more diverse range of ages, which may minimize sensitivity to censoring. Nevertheless, it will be important to fully quantify the implications of random visit times, as it is also possible that under this constraint, a single observation does not provide adequate data to inform sire ranking. That is, two or more observations may be required if the timing of observations cannot be aligned across herds.

### Estimated breed effects depended on analysis method and phenotype censoring

The breed effects, which represent the estimated difference between breeds, depended on analysis method and scenario for phenotyping. The negative Jersey breed effect solutions when all three observation visits were included (scenario EML) indicated that Jersey animals experienced BP4 elevation before Holstein–Friesian animals, although the size of this difference varied by analysis method. Furthermore, within each analysis method, the estimated breed difference was smallest when the scenario did not include the ‘early’ visit (that is, when left censoring was increased). It has been previously reported that Jersey animals attained puberty 70 d earlier than Holstein–Friesian animals in a New Zealand system [1]. A breed difference of 70 d falls within the upper end of our credibility intervals under the CAT and AUG methods when the scenarios included the ‘early’ observation; however, using the AGEVISIT method we did not estimate a plausible breed difference of up to 70 d under any scenario. Our results viewed alongside previous research indicate that the breed difference for AGEP4 is well estimated for the CAT and AUG methods when three observations are included but may become underestimated as the phenotype becomes more censored, especially left censored. Conversely, the breed difference under the AGEVISIT method may be consistently underestimated across all phenotyping scenarios.

It is important that differences between breeds are well estimated. Firstly, in a multi-breed analysis, the accuracy of breed effects will influence the dispersion parameters, and thus affect estimates of heritability. When breed is fitted as a fixed effect in a genetic analysis, the heritability should represent the proportion of phenotypic variance that is due to within breed additive genetic variance (that is, variance that cannot be attributed to residuals and relevant fixed effects, including breed). If the breed effect is poorly estimated, then variance due to breed can be incorrectly attributed to additive genetic variance, thus inflating the estimated heritability. Secondly, the inaccuracy of breed differences will systematically effect EBV rankings when animals are compared across breed. In dairy sectors with mixed-breed populations, such as in New Zealand, farmers are often provided with ‘across breed’ EBVs (that is, the fixed breed effects are added to the within-breed EBV). This allows farmers to select the highest-ranking animals on an index, or for a given trait, irrespective of breed composition. If the breed difference for a trait is not accurate, this will result in systematic under- or over-estimation of a certain breed, leading to suboptimal genetic selection decisions. Third, the estimated breed differences may be useful in optimizing future phenotype collection. For example, our results indicate that the model solutions are most sensitive to left censoring of the phenotype. The estimated breed difference can provide useful insight for reducing left censoring when measuring this trait across breed.

The Jersey breed was not well represented by pedigree Jerseys, as in this study as there were only 24 animals that were > 90% Jersey, and they were all in the same contemporary group. However, around half of the animals included in this study were admixed crosses between the Holstein–Friesian and Jersey breeds, and these cross-bred animals were present in 53 of the 54 herds. The large number of cross-bred animals provided a reasonable basis for estimating a breed difference between Holstein-Friesians and Jerseys, as around half of the phenotypes included in the analysis will contribute to the solution for this fixed covariate. As indicated, we were not able to separate the effects of heterosis and breed, as heterosis coefficients and breed fractions were correlated in this population. It is possible that if we were able to repeat this study in a population with greater representation of 100% Jersey animals, we would find that the estimation of breed differences behaved differently across method and across scenarios. Hence, further investigations including data from this breed are required.

### Sensitivity of herd effects to increased censoring depended on analysis method

We quantified the extent that herds re-rank based on mean AGEP4 across the phenotype censoring scenarios using a Pearson correlation coefficient. Our results indicated a large degree of re-ranking of herds between our control scenario and the test scenarios with increased censoring for the CAT and AGEVISIT method. In contrast, the herd rankings under the AUG method were remarkably consistent across various visit scenarios. Similar to the estimation of breed differences, the accuracy of the herd effects are important for the accurate estimation of dispersion parameters and EBVs. The stability of herd effects between scenarios for the AUG method will contribute to the stability of EBVs between scenarios.

### Heritabilities were robust to method and phenotype censoring

Estimated heritabilities for AGEP4 varied little between different methods or scenarios. We did, however, observe a tendency for higher heritabilities when the ‘early’ visit was included in the analysis. This aligns with indications from this research that breed and herd effects appear better estimated under scenarios that minimize left censoring. Including the early visit in the analysis differentiates animals with left-censored phenotypes and provides separation between their phenotypes and those of the remaining animals in the study. It would seem that this is an important distinction to make when estimating variance parameters, although the importance of including the early visit may be relative to the extent of left-censoring in the dataset. Across the methods and regimes analysed, the heritability for AGEP4 was estimated between 0.05 and 0.60, with a mean of around 0.25. This spread in heritabilities is comparable to those reported in current literature, which range from 0.10 to 0.56 [7, 20].

### General limitations

Using our method of AGEP4 phenotyping based on one to three herd visits to collect BP4, there will be some animals with false negatives for BP4 status. Concentrations of BP4 are cyclic in post-pubertal heifers and are not elevated for about one week of the three-week estrous cycle. Hence, these periods of naturally low BP4 in post-pubertal animals will have produced false negatives in our data (i.e., a pubertal animal will present with basal BP4 roughly 30% of the time). The four-week interval between herd visits means that natural BP4 depression in post-pubertal animals can only create a maximum of one false negative record per animal. False negatives will mean that some animals will be penalized incorrectly. False negatives may increase the residual variance associated with our AGEP4 phenotypes, and therefore reduce the estimated heritability of our phenotype. We would expect false negatives to occur at random across our population, without bias towards the daughters of any particular sire. Therefore, we would not expect false negatives to have implications for sire rankings. This theory is supported by the findings [21] where the authors tested the implications of phenotype censoring using AGEP4 phenotypes measured in a small population of approximately 500 cows measured weekly. In that study, AGEP phenotypes were compared under two levels of censoring. The least censored version of the phenotype involved an extended period of weekly blood testing, which would essentially eliminate the occurrence of false negatives in the phenotypes. The second, more censored version of the phenotype mirrored the phenotyping strategy that we have used in the present study. Their results indicated that phenotype censoring in this manner had minimal implications on animal EBV rankings.

The ‘control’ scenario presented here used only monthly measures and therefore was already censored, and this limits our ability to make inference about the implications of censoring. Our results indicate that the marginal gain of a third BP4 observation is not likely to justify the cost or effort associated with this measurement. The least censored version of this phenotype would be daily observations for each animal over the complete window where it was biologically possible for them to attain puberty; however, we were not able to test the implications of moving from no censoring (daily observations) to three observations per daughter. That said, Stephen et al. [6] recently investigated the implications of phenotype censoring using simulated AGEP phenotypes. In their study, the censoring scenarios mirrored most of those included in the present study (all except the E/M/L scenario), but the control scenario used uncensored daily phenotypes. The authors used a data augmentation approach and reported only minimal differences between either heritability estimates or EBVs across censoring scenarios.

## Conclusions

It may be feasible for animal breeders with a given budget to increase the number of animals measured in a progeny test by reducing the number of observations per animal when collecting performance data for time-dependent traits such as AGEP. The three main findings of our study are, first, using the three analysis methods investigated, two observations per offspring may provide comparable sire rankings to three observations per offspring. Second, using a data augmentation approach, one observation per offspring may provide comparable sire rankings to three observations per offspring. Third, using any of the three methods investigated, one or two observations per offspring may provide comparable estimated heritabilities. However, it is worth noting that breed differences tended to decrease as phenotype censoring increased, and so care should be taken when applying these findings to an across-breed evaluation system.

Our findings have implications for the design of large-scale phenotyping initiatives as reducing the number of observations per animal for time-dependent traits could lead to a larger number of animals phenotyped, potentially improving accuracy and intensity of selection.

## Data Availability

The datasets generated and/or analyzed during the present study are only available from the corresponding author on reasonable request.

## Abbreviations

AGEP: Age at puberty
AGEP4: Age at first elevation (> 1 ng/mL) in blood plasma progesterone
AGEVISIT: Age on visit method
AUG: Data augmentation method
BP4: Blood plasma progesterone
CAT: Visit category method
E: Early
E/M/L: Early, mid or late
EBVs: Estimated breeding values
EL: Early and late
EM: Early and mid
HF: Holstein-Friesian
J: Jersey
L: Late
M: Mid
MCMC: Markov-chain Monte Carlo
ML: Mid and late
N: No
XB: Cross-breed
Y: Yes
